# The KAP Evaluation of Intervention on Fall-Induced Injuries among Elders in a Safe Community in Shanghai, China

**DOI:** 10.1371/journal.pone.0032848

**Published:** 2012-03-27

**Authors:** Ling-ling Zhang, Koustuv Dalal, Ming-min Yin, De-guo Yuan, Johanna Yvonne Andrews, Shu-mei Wang

**Affiliations:** 1 Key Laboratory of Public Health Safety, School of Public Health, Ministry of Education, Fudan University, Shanghai, China; 2 Örebro University, School of Health and Medical Science, Department of Public Health Science, Örebro, Sweden; 3 Tufts University, Somerville, Massachusetts, United States of America; Federal University of Rio de Janeiro, Brazil

## Abstract

**Background:**

To evaluate the effect of an intervention on fall induced injuries of elderly people in a safe-community in Shanghai and to discuss an intervention model that is proper for the community to generalize.

**Methodology/Principal Findings:**

Five neighborhood areas in a Safe Community were purposively selected. All individuals aged 60 years or over in five neighborhoods were prospective participants. From randomly selected prospective households with elders, 2,889 (pre intervention) and 3,021 (post intervention) elderly people were included in the study. Knowledge, Attitude and Practice Model (KAP) questionnaires were used at the pre- and post-intervention phase for fall-induced injury prevention in the community. Descriptive statistics and chi-square tests were used. After the intervention, knowledge about the prevention of fall-induced injuries increased, as did attitudes, beliefs and good behaviors for fall prevention. Behavior modification was most notable with many behavior items changing significantly (p value<0.0001).

**Conclusions/ Significance:**

The integrated program for reducing fall-related injuries in the community was effective in improving fall prevention among the elderly, but the intervention still needs further improvement.

## Introduction

Fall injury is a leading cause of death and disability among older adults. The World Health Organization (WHO) estimated that around 424,000 fatal falls occur globally each year and another 37.3 million falls are severe enough to require medical attention [1]. Approximately one in every three elders (>65 years) living in a community falls. The incidence rate of falls of older adults living in a community varies from 517 to 683 per 1,000 person-years [2], [3]. Elderly falls are an expensive age related public health problem with four decades of research regarding prevention strategies [4].

Falls are a leading cause of injury death among elders in China [5], [6], [7], [8]. A study from the Shanghai Xuhui district indicates that falls account for almost two-thirds (64.57%) of the total deaths caused by unintentional injury among the elderly [9]. The WHO reported that China has the largest fall related disease burden in the world. The annual direct medical cost of falls is over 5 billion Yuan with a social cost of up to 80 billion Yuan [10]. Hence, studies aimed at fall injury prevention in China are urgently needed. Numerous studies have been conducted on multidisciplinary, multi-factorial, health and environmental risk factor screenings and interventions of elderly fall prevention [1], [2], [4]. However, literature specifically relating to China is limited to observational studies of risk factor analyses and warrants intervention [1], [11], [12].

Studies have shown that falls in the elderly are avoidable to a certain extent [13]. A recent systematic literature review indicated that environment and behavior modification are effective in preventing falls among the elderly [2]. It has been evidenced that some elderly people realized the risk of fall but they have short of knowledge on how to prevent. Some others even didn't know what could be the risk factors. It is important to let the elderly people know what are the risk factors for fall injury, what they could do to prevent them [14]. At the same time, it is being suggested in the Knowledge, Attitude and Practice Model (KAP) that knowledge is the base; belief is a motivator, while the forming and changing of behaviors is the ultimate goal [15]. Knowledge, attitude and practice are closely associated with behavior modification and are crucial for risk reduction of fall among elderly [2], [14]–[17].

With the Safe Community movement in China, Shanghai has initiated the community safety promotion program. Injury prevention and safety promotion at the community level are main objectives of the Safe Community program (www.phs.ki/csp). Shanghai has launched many initiatives for the elderly injury prevention program under the Safe Community network. In a designated Safe Community in Shanghai, the KAP model was applied as an intervention to improve the health knowledge and skills for reducing risk factors of fall-induced injuries among older adults. The intervention targeted the elderly (>60 years) and was aimed towards developing their beliefs and attitudes step by step and to thereby generate fall preventing behaviors to help reduce the risks of fall.

The current study evaluated the effect of the KAP intervention for fall injuries among the elderly in a Safe Community in Shanghai, China. Such an assessment of the community-based intervention will provide a basis for assigning an effective intervention model for reducing risk factors of the elderly fall-induced injuries, especially in low and middle-income countries.

## Methods

The study subjects were seniors aged 60 and over living in five preselected areas at a designated Safe Community in Shanghai. An intervention for fall prevention was performed in two stages, as part of the Safe Community movement. In the first stage, the awareness campaign, knowledge generation and training activities were provided. In the second stage, environment modifications for fall prevention were introduced. Household surveys at pre- and post-intervention phases were performed.

### Interventions

As part of the movement, when a Safe Community was established, various kinds of injury prevention and safety promotion campaign materials, including fall prevention were distributed without cost to the residents. A safety education and training program was launched for older residents. An accidental injury prevention knowledge training network was set up within the community. A series of lectures underlying the prevention of fall-related injury among elderly was held regularly and a psychological counseling room named “red sunset” was opened for necessary support to the elderly. Professionals provided training for balance exercises for the elderly. As part of the environment modification, the elder's home environments and the design of outdoor exercising locations were improved. Environment modification includes installation of door and night light, installation of handrail in bathroom and stair, using skid proof mat in bathroom, and others which can reduce the risks of fall. Personal knowledge such as whether the elderly persons know that regular exercise, appropriate size of shoes can reduce risk of or prevent fall injuries. Behavior modification such as how do the elderly persons have practiced the referred training in their daily life. Actual variables for knowledge, attitude and beliefs and behavior are mentioned at tables 1, 2 and 3. More details of the intervention are available elsewhere or from the authors [18], [19].

**Table 1:** Comparison of knowledge of fall-induced injuries between pre- and post-intervention phases.

	Pre-intervention	Post-intervention	χ2	P value
	No. responded ‘Yes’ (% of N)	No. responded ‘Yes’ (% of N)		
1. Fall-induced injuries are the leading causes of hospital admission and disability	2161(76.15)	2175 (75.49)	0.33	0.57
2. Regular exercise can reduce the chance of falling	2285(80.51)	2482 (85.79)	28.35	<0.0001
3. When falling down, you should relax and bend your body then yield yourself to roll	1736(61.17)	1775 (62.28)	1.36	0.24
4. Right-sized clothes can reduce the chance of falling	1907(67.20)	2037 (71.03)	9.80	0.0017
5. Under favorable circumstance with bright lights and enough lighting, the risk of falls can be lowered	2588(91.19)	2631 (90.38)	55.20	<0.0001
6. Old people should choose sofa of low-softness	2531(89.18)	2574 (89.44)	3.93	0.14
7. Old people should choose carpet of soft fabric with its edges being fixed	947(33.37)	1194 (40.97)	46.52	<0.0001
8. The house of old people should be properly designed and free of hazards	1924(67.79)	2129 (71.25)	9.73	0.02

**Table 2:** Comparison of attitude and beliefs about fall-induced injuries between pre- and post-intervention phases.

	Pre-intervention	Post-intervention	χ2	P value
	No. responded ‘Yes’ (% of N)	No. responded ‘Yes’ (% of N)		
1. My health status is still good	565 (19.91)	479 (17.17)	17.55	0.0005
2. I enjoy doing exercise	1776 (62.58)	2058(71.88)	56.01	0.000
3. The safety of my house is very good	589 (20.75)	504 (17.46)	75.06	0.000
4. It isn't possible for me to fall down and get injured	235 (8.28)	180 (6.28)	30.43	0.000
5. I don't worry about falling down and getting injured	719 (25.33)	678 (24.01)	1.34	0.25
6. I'm weak and need to participate in fall-intervention activities	1709 (60.22)	1821 (65.04)	13.97	0.000
7. Carrying out knowledge training program on fall-induced injury in the community is of great necessity	1247 (43.94)	1417 (51.43)	35.34	0.000
8. Falls can be completely prevented if appropriate methods are adopted	2010 (70.85)	2841 (72.85)	39.89	0.000
9. I can do a lot to lower the possibility of injury	1152 (40.59)	1170 (41.76)	0.88	0.64
10. I am sure I can get enough information to prevent fall-induced injury	1790 (63.07)	1964 (70.37)	33.72	0.000
11. I'd like to participate in fall-intervention activities	2358 (83.09)	2391 (84.28)	1.48	0.22
12. I can complete the task assigned to me quickly when participating in fall-intervention activities	2566 (90.42)	2584 (91.63)	2.56	0.11
13. It makes me feel confident participating in fall-intervention activities	2629 (92.64)	2682 (94.34)	6.75	0.009
14. The Safe-community project can bring benefits to the lives of old adults	2777 (97.85)	2796 (97.90)	0.02	0.899

**Table 3:** Comparison of behaviors about fall-induced injuries between pre- and post-intervention phases.

	Pre-intervention	Post-intervention	χ2	P value
	Number responded ‘Yes’ (% of N)	Number responded ‘Yes’ (% of N)		
1. Exercise regularly	2222 (78.29)	2396 (84.54)	36.61	0.000
2. Doing exercise one time or more per week	2152 (75.85)	2280 (85.01)	73.08	0.000
3. Grab the handrail when going up and down stairs	2113 (74.45)	2177 (76.28)	2.55	0.11
4. Install safe handles and railings in bathroom	1040 (36.65)	1361 (48.11)	76.25	0.000
5. Use anti-skid material on the floor of bathroom and base of the bathtub	1188 (41.86)	1556 (55.79)	109.25	0.000
6. Check the soles whether they are skid-free	2359 (83.12)	2552 (89.86)	55.15	0.000
7. Check the lights in the corridors and stairways	2427 (85.52)	2581 (91.30)	46.15	0.000
8. Consult the doctor whether the drugs prescribed will influence balance	1347 (47.46)	1558 (55.43)	35.84	0.000
9. Have eyes checked by eye doctor	1731 (60.99)	1792 (64.21)	1.83	0.18

### Study procedure

Two household surveys were conducted in December 2008 (pre-intervention) and in the same month during 2009 (post-intervention) by the trained investigators using pre-tested questionnaires.

Initially a pilot study was performed to pre-test the questionnaire and finalize the interview procedure including interviewers training. Initially, fifteen elderly persons were invited to answer the questionnaire to judge whether they can understand the questions, whether they understand correctly, whether they can correctly answer the question based on the instruction. Based on this some modifications were made and the instruments and procedure were finalize. In total, 20 interviewers were trained for the study. Two to four interviewers were trained to conduct the household survey in each study area. Interviewers were from the neighborhood committee with at least a high school (12+ years) education. All interviewers were familiar with the neighborhood and were knowledgeable about the neighborhood welfare system. They received training from professional experts and they passed performance appraisal which included ethical issues.

The Assessment form for fall-related knowledge, attitude and practice (K-A-P) among elderly in community was used [15]. Questions were dichotomous (Yes / No) and easy to respond to. Initially, the questionnaires were set in the English language. However, Chinese language versions of the questionnaires were also used. English-Chinese translation was verified by the ‘translation and back-translation’ method by the lead researchers (Associate Professor rank).

Initially, a general notice was distributed to all households indicating the intention of the study. A request was made to all the elderly in the study area to participate in the study so that the fall prevention interventions could be evaluated. The interviewers visited households, identified the elderly and again explained the purpose of the study. Participants were informed that they would be followed up one year after intervention. All ethical formalities were explicitly mentioned.

**Figure 1:**
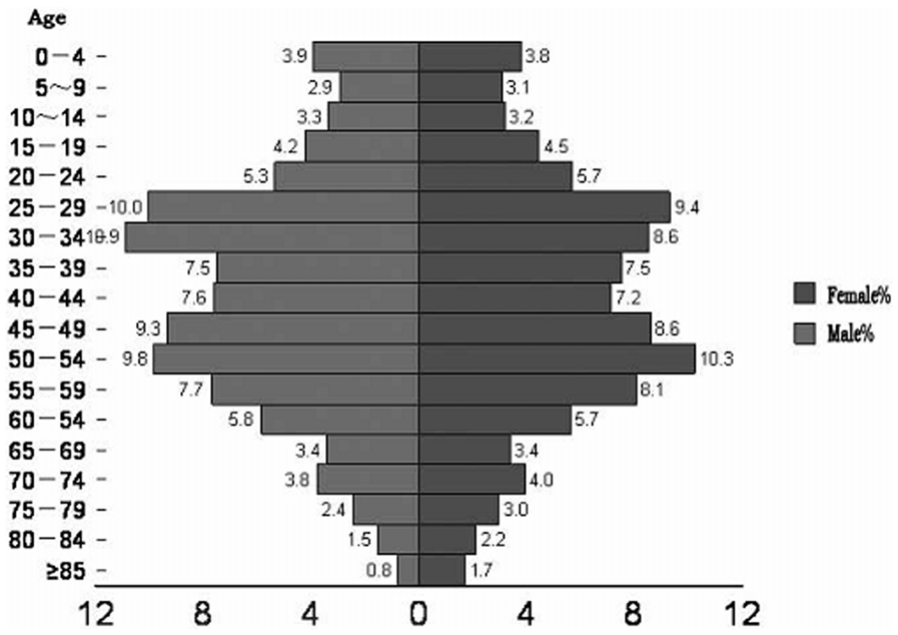
Population distribution of the five neighborhood in a Safe Community in Shanghai, in 2007.

In the selected five neighborhood areas of a Shanghai Safe Community, all the prospective households (with elderly aged >60 years) were first identified using the residential registration information. Population distribution statistics from 2007 (Figure 1) were used to identify the prospective households. Then researchers randomly selected 3,000 elderly households for interviewing the study subjects. Trained interviewers visited those selected elderly households and interviewed the elders. Out of the visited households, only 11 households were not occupied. If there were two elders in one household then both were interviewed. The response rate was more than 96% in both occasions. However, during follow up study in 2009, 3,021 elderly persons were interviewed. In 2008 few elderly who aged 59 years became 60 years in 2009 and were included in the study.

### Quality control

Before beginning the interviews, interviewers, quality control staff and researchers went through all the study procedures once more in order to apply a standard investigation methodology. Data feeding, cleaning and monitoring was performed on a daily basis to help ensure better quality control and to guarantee the authenticity and integrity of the data. Consistency tests and logic tests were performed alongside data entry. For utmost quality control, five percent of the subjects were randomly selected for re-investigation by the researchers. After, all of the data was gathered such selection and re-investigation was performed. The same group of study personnel repeated the procedures the following year (2009). The entire investigation process was monitored by the Safe Community office and by the researchers. Data collections consisted of baseline measurements and a one year follow-up survey and these were analyzed by using SAS version 9.1.3 for Windows.

### Ethical permission

Written consents were obtained from the respondents. Rights to withdraw and autonomy of the respondents were explained. The study received ethical permission from the ethical committee of the Fudan University, China ( IRB00002408&FWA00002399).

### Statistical analysis

Descriptive statistics were used for describing demographics. Chi-square tests were used to evaluate the KAP significance. A significant level p<0.05 was employed.

## Results

Initially, a total of 2,889 elderly persons were interviewed in 2008. After screening and cleaning the raw data sets, 2,838 interviews were included in the study (98.2% valid). In 2009, 3,021 elders were interviewed. In total, 2,988 questionnaires were valid (97.9% valid) and were included in the study. At the baseline survey, the mean age of elderly adults was 68 years, 48.0% of participants were male, 61.1% had a junior high school education and about 94.3% of elders were retired.

### Knowledge of fall-induced injuries

In table 1, the aspect of cognition, compared with cognition of pre-intervention, showed a significant improvement in items concerning the benefits of exercise, the importance of good lighting, the option of carpet in the living room and hazard free housing. Yet further improvement was still needed and other aspects did not show statistically different changes.

### Attitudes and beliefs

Table 2 shows a clear increase and a statistically significant difference in the percentage of elders who hold that “appropriate measures can completely prevent fall-induced injuries; it is necessary to carry out a knowledge training program on fall-induced injuries; I am confident about my access to information and I enjoy exercising; I need to participate in fall-related interventions”. Additionally, there was a significant decrease in the percentage of elders who hold that “my health status is still good; the environment of my house is safe and it isn't possible for me to fall down”. However, the change is not evident in other aspects in relation to attitudes and belief.

### Behaviors

As shown in table 3, fall-related behaviors were evaluated, since regular exercise is beneficial in improving elders' ability to cope with risk factors in the environment and for lowering the risk of fall-induced injuries (through strengthened upper and lower limbs muscles, balance-keeping and coordination abilities). The results show a significant increase in the percentage of elders who joined in the regular exercise (78.29% vs. 84.54%, p<0.05). After the intervention, the weekly exercise frequency, the installation of anti-skid home facilities and fall-preventing behaviors also significantly improved.

## Discussion

After the implementation of the intervention in the community, there were some improvements in fall-related knowledge, beliefs, attitudes and behaviors. The percentage of participant awareness about fall-related knowledge in most items reached 60% and more. The findings are better than those reported by earlier studies [20]. Therefore the findings of the current study would suggest that fall prevention interventions within Safe Communities is successful and works better than small location-pocket based intervention/s [20], [21]. This study found a modest change in the elders' beliefs about fall related risk factors and prevention. On the one hand, they realized that they have become a population with a high risk of fall as a result of increasing age, gradual physical function degradation and many risk factors in the home environments. On the other hand, they began to accept that fall-induced injuries are preventable and that there are benefits of exercise in reducing the incidence of injury and improving life quality, especially in a Safe Community.

Among the changes in the three aspects studied, the most significant change was seen in the fall-related behaviors. After the intervention, the number of elders who regularly exercised, increased meaningfully, with a statistical difference in every item except for “grab the handrail when going up and down stairs” and “checking eyesight”. These show that the integrated intervention aiming at decreasing risk factors worked well and had obvious effects. Not only did the intervention promote the elders' cognition, but it also contributed to the formation of healthy behaviors.

However, the improvement was not so noticeable in certain aspects, for example knowledge. The elders' cognition with regard to choosing the right kind of carpet was not substantial, with the percentage of elders choosing the right carpet only increasing 7.6% compared with that of pre-intervention. Also, no improvement was seen in the “fall-related injuries are the leading cause of hospital admission and disability of elders” aspect. This reminds us to not only emphasize the instruction of fall-related professional skills, but also to consider how to increase the elders' awareness of the severity of the consequences of fall in our health education in future [18]. Although the percentage of elders who held positive convictions, “I can do much to lower the possibility of injury”, increased after the intervention, it was still less than 50%, a percentage similar to the findings of Xia et al [14].

However, the current analysis revealed few important points. More emphasis was placed on how to prevent the falls caused by external risk factors for elders in the intervention group, while changing of elders' own internal factors to reduce falls was barely touched upon. Hence, the elders may lack knowledge in their efforts to reduce the risk of falls. As a result of physical degradation (muscular atrophy and blurred vision), elders have such poor control over the surroundings that they may lack confidence in their own efforts to reduce the risk of falls [22]. Most of the elderly have experienced more than one fall, and their negative convictions are reinforced to some extent. All of these indicate that more focus should be placed not only on publicity of external risk factors for falls, but also internal factors. Not only should the intervention stress the improvement of household [23] and community environments to maximize the reduction of accidental falls caused by external risk factors, but there should also be psychological counseling, encouragement and support to help the elders restore belief in a positive future.

Employing the KAP theory has shown that knowledge is the base and pre-requisite of formation of behavior, and the transformation of knowledge to behavior is influenced by many factors. It is an extremely long and complex process [15] from the acquisition of knowledge, to the cultivation and confirmation of new behaviors. After the intervention, some behaviors slightly improved, for instance, the number of the elders who installed safety handles in the bathroom. The number of those who did install safety handles is still small and this small number is consistent with other domestic research findings [20], [24]. This indicates that the intervention should be better targeted and should increase the training on fall-related knowledge. The intervention should encourage the elders' transition from knowledge and beliefs to healthy behaviors. Further discussion is still needed to determine whether there are more complex explanations.

A limitation of the study is the use of self-reports. With self-reports, recall bias and reliability can be problematic, especially in elders when they are asked about past events or when they are sensitive to the questions. Additionally, the source of the sample is relatively concentrated, as the subjects were all from the same safe community and the study did not select another community as a randomized control group. Statistical validation and reliability test of the instruments were not performed for the current study. However, the instruments were absorbed from the tested version of a recently published book [15] and also modification and finalization were performed through the pilot-study.

It should be noticed that when the elderly people have more power in improving the situation by themselves the better outcomes came such as doing exercise regularly. While when some extra resources needed neither the baseline situation nor the post-intervention situation provide satisfactory findings such as installation of handrail in bathroom. Since the safe community project is a local government endeavor the more attention can be paid to environment improvement in this regard. It is a useful KAP model which can be very helpful when carrying out elderly fall injury prevention program at community level.

Fall prevention programs are generally of low cost, effective and sustainable [14]. However, in future community interventions, the projects should consider the elders' special physiological characteristics in order to meet their needs. Also, these types of interventions should improve the community on the basis of the original implementation in order to maximize and continue the positive effects it may have on the community.

In conclusion the study suggests that integrated program for preventing fall-related injuries in the community was effective in improving fall prevention among the elderly. However the intervention needs further improvement with specific target group. Safe Community program is effective for improving KAP of the elderly population for reducing fall-related injuries which was also supported by their beliefs.
